# Impact of Age on Patient-Reported Outcomes Following Stereotactic Body Radiation Therapy for Prostate Cancer

**DOI:** 10.7759/cureus.13780

**Published:** 2021-03-09

**Authors:** Abigail Pepin, Monica Pernia, Malika T Danner, Marilyn Ayoob, Thomas M Yung, Siyuan Lei, Brian T Collins, Suy Simeng, Nima Aghdam, Sean P Collins

**Affiliations:** 1 Department of Radiation Oncology, George Washington University School of Medicine and Health Sciences, Washington, USA; 2 Department of Geriatrics, George Washington University, Washington, USA; 3 Department of Radiation Medicine, Georgetown University Hospital, Washington, USA; 4 Department of Radiation Oncology, Beth Israel Deaconess Medical Center, Harvard Medical School, Boston, USA

**Keywords:** quality of life, prostate cancer, sbrt, cyberknife, epic, elderly, survivorship

## Abstract

Purpose

Stereotactic body radiation therapy (SBRT) delivers large radiation doses to the prostate while minimizing exposure to adjacent normal tissues. Large fraction sizes may increase the risks of functional decrements. Elderly men may be at an increased risk of these toxicities due to poor baseline function and hence limited reserve. This study describes patient-reported outcomes following SBRT for clinically localized prostate cancer in the elderly.

Methods

Between 2007 and 2017, 179 hormone-naive elderly patients (≥ 70 years old) and 210 patients under 70 years old with clinically localized prostate cancer were treated with 35-36.25 Gy SBRT in five fractions utilizing the CyberKnife Radiosurgical System (Accuray Inc.). Quality of life (QOL) was assessed using the Expanded Prostate Index Composite-Short Form (EPIC-26) questionnaire at baseline and at 1, 3, 6, 12, 18, 24, 30, and 36 months following the completion of treatment. EPIC scores range from 0 to 100, with lower values representing worsening symptoms.

Results

EPIC scores in the elderly cohort mirrored those in the younger cohort. EPIC urinary obstructive/irritative scores declined at one month post-SBRT (mean change from baseline ≥70: -7.9; <70: -11.1) before returning to baseline at three months post-SBRT (mean change from baseline ≥70: -0.4; <70: -1.4). The EPIC urinary incontinence scores declined slowly over the three years following treatment without recovery (mean change from baseline ≥70: -6.6; <70: -4.8). EPIC Bowel scores transiently declined at one month post-SBRT (mean change from baseline ≥70: -8.5; <70: -9.1) and then experienced a second more protracted decline over the next three years without recovery (mean change from baseline ≥70: -4.5; <70: -1.8). Hormonal EPIC scores were not impacted by radiation treatment or age. Older men had lower baseline and post-treatment EPIC sexual summary scores at all time points. However, there was no clinically significant difference in the EPIC sexual bother score between younger and older men at baseline and following treatment.

Conclusions

In the first three years following treatment, the impact of SBRT treatment on patient-reported outcomes was minimal. Our findings suggest that SBRT for clinically localized prostate cancer should not be deferred in older men solely due to concerns of increased morbidity. Further studies should be conducted to evaluate the impact of age on outcomes or morbidity following SBRT.

## Introduction

Prostate cancer is a common malignancy in elderly men (≥70 years old). With many modalities available to treat prostate cancer, the primary goal for any definitive treatment should balance survival outcomes with quality of life (QoL) measures. In recent years, advances in radiation therapy have allowed higher radiation doses to be delivered to the prostate while sparing surrounding organs, hence reducing toxicity. Stereotactic body radiation therapy (SBRT) has gained favor due to its enhanced accuracy, intrafraction image guidance, and decreased treatment margins [1]. This decreases the uncertainty of the location of the prostate and allows treatment to be delivered with a smaller clinical target volume (CTV) to planning target volume (PTV) expansion. Furthermore, due to low alpha/beta ratio in prostate cancer, there is a potential therapeutic benefit of using larger fractionated doses. Emerging data from randomized trials suggest that SBRT may provide similar clinical outcomes to other radiation modalities with low rates of high-grade toxicity [2]. Multi-institutional health-related QoL data suggest that SBRT is well tolerated overall at long-term follow-up [3].

Although elderly men are more likely to be diagnosed with unfavorable prostate cancer, they are less likely to receive local therapy [4-6]. The current National Comprehensive Cancer Network (NCCN) treatment guidelines base treatment decisions using 10- and 20-year life expectancy predictions. However, age by itself is a poor proxy for life expectancy. The European Association of Urology guidelines recommend that life expectancy, overall health status, and tumor characteristics should factor into treatment decisions [7]. The concern that overtreatment of prostate cancer in elderly men can increase treatment-related side effects remains.

Elderly men may be a population “at risk” of treatment-related functional decline [8]. The etiology is not clear, but it may be due to concerns about excessive toxicity in elderly men due to decreased pretreatment function [8]. Some studies have reported similar rates of acute and long-term side effects among different age groups in men undergoing radiotherapy [9-11]. Other reports have suggested that elderly men experienced worsening sexual function, bowel function, and urinary function following external beam radiation therapy (EBRT), while others have suggested no significant impact on urinary function, sexual function, or sexual bother QoL measures [9,12-14].

Although several studies investigating QoL in elderly men treated for prostate cancer have been reported, long-term QoL outcomes in elderly men following SBRT are largely unknown. The goal of this study is to report on long-term QoL outcomes in elderly men undergoing SBRT for treatment of their prostate cancer.

Portions of this research were presented at the International Society of Geriatric Oncology (SIOG) 2020 Annual Meeting Online, October 1, 2020.

## Materials and methods

Patient selection

The Georgetown University Institutional Review Board (IRB) approved this single-institution retrospective review of prospectively collected data. All individuals diagnosed with localized prostate cancer who received SBRT at MedStar Georgetown University Hospital from 2007 to 2017 were eligible for inclusion. Individuals were required to have a minimum follow-up of 36 months post-SBRT to be included in this study. Individuals treated with androgen deprivation therapy were excluded from the study.

SBRT treatment planning and delivery

Simulation, contouring, and treatment planning were performed based on an institutional protocol [15]. SBRT was delivered using the CyberKnife robotic radiosurgical system (Accuray Inc., Sunnyvale, CA). Gold fiducials were placed into the prostate using ultrasound guidance. Fused thin-cut CT images and high-resolution MR images were used for treatment planning. The CTV included the prostate and proximal seminal vesicles. The PTV included a 3 mm (inferior, superior, and posterior) or 5 mm (anterior and lateral) expansion around the CTV. Fiducial-based tracking was used to account for interfraction and intrafraction prostate motion. Patients were treated with 35 or 36.25 Gy of radiotherapy delivered in five fractions of 7-7.25 Gy each to the PTV. The prescription isodose line was limited to ≥75% to restrict the maximum prostatic urethra dose to 133% of the prescription dose.

Follow-up and statistical analysis

Urinary, bowel, hormonal, and sexual function and bother were prospectively assessed using the Expanded Prostate Index Composite (EPIC)-26 at baseline (one to two hours prior to the first fraction), every three months for the first year post-treatment, every six months for the second year, and then yearly thereafter. The urinary domain was divided into obstructive/irritative and incontinence subdomains due to their varying patterns following treatment. Bowel, hormonal, and sexual domain scores were calculated by obtaining an average across each time point. Sexual domain was also divided into sexual bother and sexual function domains.

To discriminate the impact of age on QoL, the patients were divided into two age groups: <70 years old (n =210) and ≥70 years old (n = 179). Paired t-tests were used to statistically compare changes between time points from baseline. Chi-square analysis was used to determine demographic differences between the cohorts. EPIC scores for the bowel domain and its individual questions range from 0 to 100, with lower values representing worsening bowel symptoms. The minimally important difference (MID) in EPIC score was defined as a change of one-half standard deviation (SD) from the baseline [16].

## Results

From 2007 and 2017, 389 prostate cancer patients were treated per our institutional SBRT protocol on a prospective QoL trial (Table 1).

**Table 1:** Patient characteristics and treatment

	All Patients (n=389)	Percentage of Patients < 70 years (n=210)	Percentage of Patients ≥ 70 years (n=179)	p-Value
Age (years)	Median: 69 (42-94)	Median: 64 (42-69)	Median 73: (70-94)	
Race				
White	54.2% (211)	46.7% (98)	63.1% (113)	0.0008
Black	38.0% (148)	46.7% (98)	27.9% (50)	
Other	7.7% (30)	6.7% (14)	8.9% (16)	
Charlson Comorbidity Index				
0	56.2% (219)	59.0% (124)	53.1% (95)	0.4503
1	26.2% (102)	25.2% (53)	27.4% (49)	
>2	17.5% (68)	15.7% (33)	19.6% (35)	
Risk group (D’Amico)				
Low	33.4% (130)	39.5% (83)	26.3% (47)	0.0052
Intermediate	60.9% (237)	57.1% (120)	65.4% (117)	
High	5.7% (22)	3.3% (7)	8.4% (15)	
Treatment dose				
35	25.2% (98)	21.9% (46)	29.1% (52)	0.1057
36.25	74.8% (291)	78.1% (164)	70.9% (127)	

A total of 210 patients were <70 years old and 179 patients were over ≥70 years old. They were ethnically diverse, with 54.2% being of Caucasian ancestry with a median age of 69 years (range: 42-94 years). The elderly men in our study were more likely to be Caucasian. By D’Amico classification, 33.4% patients were at low risk, 60.9% were at intermediate risk, and 5.7% were at high risk. Elderly individuals were more likely to present with high-risk disease than their younger counterparts (8.4% vs. 3.3%). Approximately 75% of the patients were treated with 36.25 Gy in five fractions of 7.25 Gy each.

**Figure 1:**
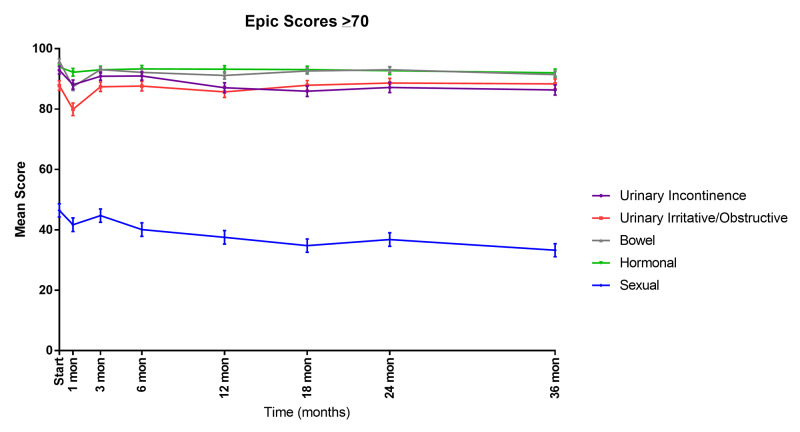
Mean EPIC-26 scores for urinary irritative/obstructive, urinary incontinence, bowel, hormonal, and sexual domains in men ≥ 70 years old Errors bars represent 95% CI. EPIC-26, Expanded Prostate Index Composite-Short Form

Urinary, bowel, and hormonal domain scores remained high during follow-up in the elderly cohort (Figure 1). The EPIC urinary obstructive/irritative scores declined (mean change: -7.9 from baseline) at one month after treatment before recovering to baseline at three months (mean change: -0.4 from baseline). The urinary incontinence scores slowly declined in the three years post-SBRT (mean change: -6.6). This change was statistically significant (p <0.05) but not clinically significant (MID: 8.8). Bowel scores clinically and significantly declined at one month post-SBRT (mean change: -8.5; p<0.05; MID: 6.4). Bowel scores then experienced a more protracted decline over the next three years without recovery, which was not clinically significant (mean change: -4.5; p<0.05; MID: 6.4). Across every time point, sexual scores remained lower than any other domain.

**Figure 2:**
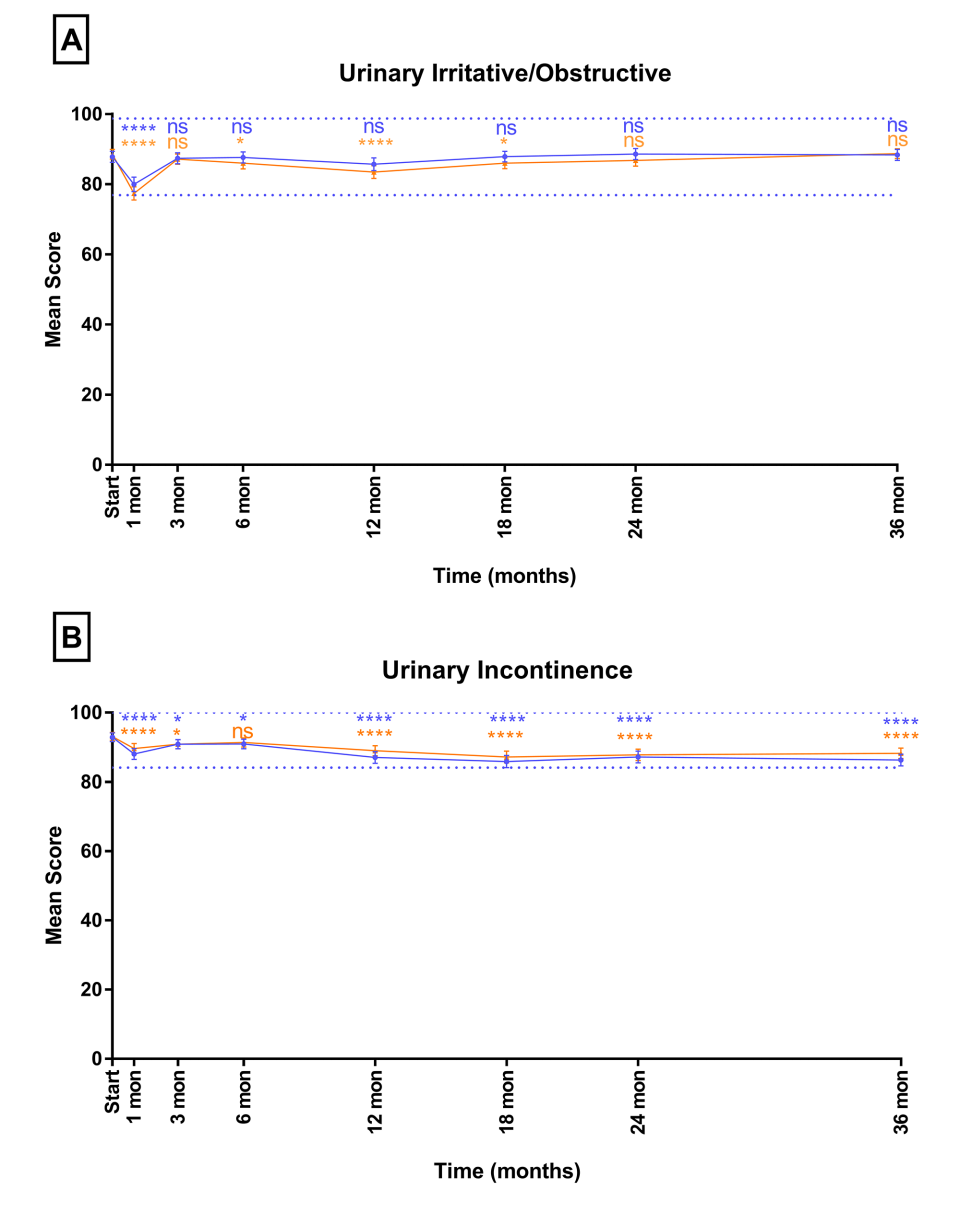
Mean EPIC-26 scores for (a) urinary irritative/obstructive and (b) urinary incontinence in men <70 years old (blue) and ≥70 years old (orange) Dashed lines represent 1/2 SD above and below baseline for the elderly cohort. Error bars represent 95% CI. *p-value < 0.05. ****p-value < 0.0001. EPIC-26, Expanded Prostate Index Composite-Short Form; ns, nonsignificant

We sought to compare the elderly cohort to the younger cohorts. Mean urinary irritative/obstructive and urinary incontinence EPIC scores over time are shown in Figure 2. The elderly cohort experienced similar trends in the younger cohort in the urinary irritative/obstructive domain (Figure 2A). In both cohorts, the EPIC scores declined at one month post-SBRT. This decline was statistically significant (p<0.05) in both cohorts. The decline was clinically significant in the younger cohort, but it was not clinically significant in the elderly cohort (mean change from baseline ≥70: -7.9, MID: 10.9; mean change from baseline < 70: -11.1, MID: 10.4). For both groups, domain scores recovered to baseline by three months and were maintained to 36 months (mean change from baseline ≥70: +0.5, MID: 10.9; mean change from baseline <70: +0.24, MID: 10.4). Likewise, the elderly cohort experienced similar trends to the younger cohort in the urinary incontinence domain (Figure 2B). Similar to the elderly cohort, the younger cohort experienced a statistically significant decline at one month post-SBRT (p<0.05). This decline was not clinically significant for either cohort (mean change from baseline ≥70: -6.6, MID: 8.8; mean change from baseline <70: -4.8, MID: 8.8).

**Figure 3:**
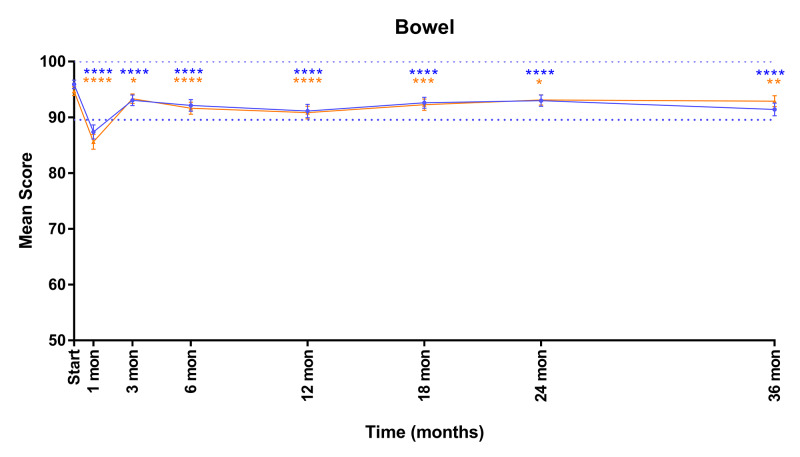
Mean EPIC-26 scores for bowel domain in men <70 years old (blue) and ≥70 years old (orange) Dashed lines represent 1/2 SD above and below baseline for the elderly cohort. Error bars represent 95% CI. *p-value < 0.05. **p-value < 0.01. ***p-value < 0.001. ****p-value < 0.0001. EPIC-26, Expanded Prostate Index Composite-Short Form

Mean EPIC bowel scores over time are shown in Figure 3. In both cohorts, EPIC Bowel scores transiently declined both clinically and statistically significantly at one month post SBRT (mean change from baseline ≥70: -8.5, MID: 6.4; mean change from baseline <70: -9.1, MID: 7.3) and then experienced a more protracted but nonclinically significant decline over the next three years without recovery (mean change from baseline ≥70: -4.5, MID: 6.4; mean change from baseline <70: -1.8, MID: 7.3).

**Figure 4:**
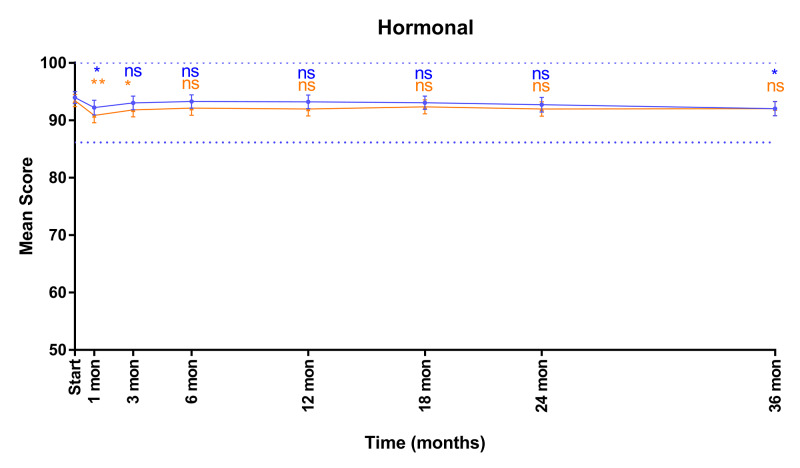
Mean EPIC-26 scores for hormonal domain in men <70 years old (blue) and ≥70 years old (orange) Dashed lines represent 1/2 SD above and below baseline for the elderly cohort. Error bars represent 95% CI. *p-value < 0.05. **p-value < 0.01. EPIC-26, Expanded Prostate Index Composite-Short Form; ns, nonsignificant

Mean EPIC hormonal domain scores are shown in Figure 4. Hormonal scores acutely declined at one month post SBRT (p<0.05; mean change from baseline ≥70: -1.7, MID 7.8; mean change from baseline <70: -2.6, MID: 8.8) before recovering to baseline at three months. Scores were maintained to 36 months post-SBRT (≥70 change: -1.9, MID 7.8; <70 change: -1.4, MID: 8.8).

**Figure 5:**
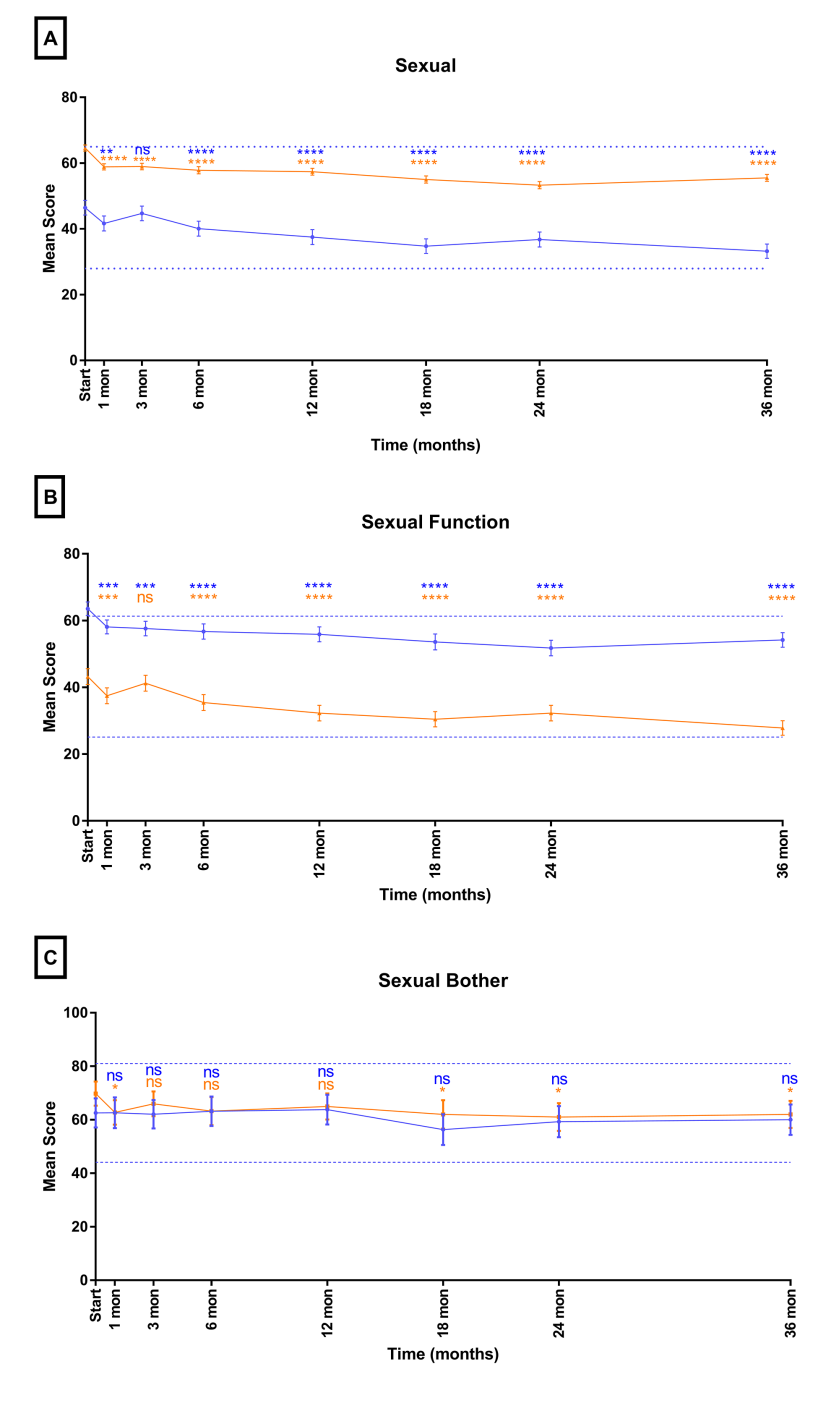
Mean EPIC-26 scores for (a) overall sexual domain, (b) sexual function domain, and (c) sexual bother domain in men <70 years old (blue) and ≥70 years old (orange) Dashed lines represent 1/2 SD above and below baseline for the elderly cohort. Error bars represent 95% CI. *p-value < 0.05. **p-value < 0.01. ***p-value < 0.001. ****p-value < 0.0001. EPIC-26, Expanded Prostate Index Composite-Short Form; ns, nonsignificant

Mean EPIC sexual domain scores are shown in Figure 5a, with sexual bother shown in Figure 5b and sexual function shown in Figure 5c. The baseline overall sexual domain scores were 64.6 and 46.4 in the younger and elderly cohorts, respectively. The sexual domain scores statistically significantly declined in the 36 months following treatment (≥70 change: -13.2; <70 change: 9.1). These declines were not clinically significant (≥70 MID: 18.5; <70 MID: 17.0). The baseline sexual function scores were 43.2 and 63.5 in the elderly and younger cohorts, respectively. In the elderly cohort, the men experienced a decline from 43.2 at start of treatment to 27.8 at 36 months post-SBRT (change: -15.4; MID: 18.1). In the younger cohort, the men also declined from 63.5 to 54.2 in the same timeframe (change: -9.3; MID: 17.0). By comparison, baseline sexual bother scores were 62.6 and 69.8 in the elderly and younger cohort, respectively. The sexual bother score declined 2.5 points in the elderly cohort and 7.7 points in the younger cohort, respectively (≥70 MID: 18.5; <70 MID: 16.6).

## Discussion

Health-related QoL measures of urinary function, urinary bother, and bowel function in patients who underwent radical prostatectomy (RP) and EBRT have been reported in the elderly. Similar to our cohort, a Japanese study reported that patients who underwent definitive EBRT for the treatment of their prostate cancer reported no significant changes with regard to general or disease-specific health-related QoL measures with the exception of sexual function [17]. By comparison, elderly men who underwent radical prostatectomy were noted to have significant declines in urinary function with high rates of urinary incontinence [5,17,18]. Similar to prostate cancer patients who have undergone conventionally fractionated EBRT or brachytherapy, urinary incontinence scores were high three years following SBRT in this series [19,20].

Our elderly cohort treated with SBRT reported similar QoL outcomes to the younger cohort in the urinary incontinence, urinary irritative/obstructive, bowel, and hormonal domains. In all these domains, both cohorts experienced an acute decline one month post-SBRT treatment, which recovered to baseline by three months post-SBRT treatment. In all domains, with the exception of the urinary irritative/obstructive and hormonal domains, a gradual decline was seen in both cohorts that was not clinically significant, but it was statistically significant in the urinary incontinence and bowel domains (p<0.05).

In our current series, we demonstrated that the sexual domain had the most pretreatment difference between our elderly population and the population under 70 years of age. In particular, elderly individuals demonstrated a poor baseline sexual function. This pretreatment difference was not demonstrated in the sexual bother domain. However, in terms of sexual function, the younger cohort experienced less of a decline in sexual function (change: -9.3) than did the elderly cohort over time (change: -15.4) and ended at a higher 36-month post-SBRT mean score than did the elderly cohort (36.6 vs. 27.8). These findings are consistent with previously reported literature suggesting that younger age and better pretreatment sexual functioning were associated with a higher probability of functional erections two years after EBRT [21]. Age-related changes to sexual function are difficult to differentiate from treatment-related decrements. However, previous literature has noted that older men may have a faster functional decline than their younger counterparts, consistent with our findings in the current study [12].

Older patients are more likely to have comorbidities [5]. Burden of comorbid illness is strongly associated with pretreatment sexual, urinary, bowel, and hormonal dysfunction [8]. The prevalence of comorbid illness in our patient cohort was high. Interestingly, the mean Charlson Comorbidity Index for our elderly cohort was 0.715 compared to 0.738 in the younger cohort. The difference in comorbidity between the two cohorts was not significant (p>0.05). Frequently, the decreased use of curative intent treatments is likely secondary to perceived increased treatment-related morbidity and health-related QoL concerns in this population due to comorbidities and age [5,6]. As such, elderly patients with prostate cancer are more likely to undergo observation compared to younger populations [5,17]. Our study demonstrates that SBRT was well tolerated in the elderly cohort with high levels of comorbidity.

This study has several limitations. Frequently, age-related changes can be hardly distinguished from treatment-related changes. The patient population was derived from a single-institution cohort that can limit the translation of our work to the general population. The retrospective nature of this analysis also places limitations on the current work. Furthermore, the cohorts were unbalanced, with the elderly cohort being significantly more likely to be Caucasian, a population which has been shown to have better QoL and high-risk disease compared to their younger counterparts. This has several implications on our study. With regard to race, elderly African American men exhibited a significant worsening of their sexual domain over time compared to the Caucasian cohort (p<0.05), though this difference was not demonstrated in individuals <70 years of age (p>0.05). Although elderly patients were more likely to have high-risk disease, individuals included in this study did not undergo hormone therapy, and there was no significant difference between radiation dose administered between the elderly and younger cohorts.

## Conclusions

In the first three years, the impact of age on patient-reported outcomes was minimal. Patient-reported urinary, bowel, and hormonal domain scores remained high in the three years following treatment in our elderly cohort. QoL outcomes were similar between our elderly patients and our patients under 70 years of age in the urinary incontinence, urinary irritative/obstructive, bowel, and hormonal domains. The elderly cohort demonstrated poor baseline and post-treatment sexual function in comparison to the younger cohort, a difference that was not demonstrated in the sexual bother domain. SBRT for clinically localized prostate cancer should not be deferred in older men solely due to concerns of increased morbidity. Further studies should be conducted to evaluate the impact of age on outcomes or morbidity following SBRT.
